# Dihydrochalcones with Antiinflammatory Activity from Leaves and Twigs of *Cyathostemma argenteum*

**DOI:** 10.3390/molecules18066898

**Published:** 2013-06-10

**Authors:** Jariya Somsrisa, Puttinan Meepowpan, Samroeng Krachodnok, Haruthai Thaisuchat, Sittiporn Punyanitya, Narong Nantasaen, Wilart Pompimon

**Affiliations:** 1 Laboratory of Natural Products, Faculty of Science and Center for Innovation in Chemistry, Lampang Rajabhat University, Lampang 52100, Thailand; E-Mails: somsrisa@windowslive.com (J.S.); krachodnok@lpru.ac.th (S.K.); haruthaithai@yahoo.com (H.T.); 2 Department of Chemistry, Faculty of Science and Center for Innovation in Chemistry, Chiang Mai University, Chiang Mai 50300, Thailand; E-Mail: pmeepowpan@gmail.com; 3 Department of Surgery, Faculty of Medicine, Chiang Mai University, Chiang Mai 50300, Thailand; E-Mail: punyanitya@hotmail.com; 4 The Forest Herbarium, Department of National Park, Wildlife and Plant Conservation, Ministry of Natural Resources and Environment, Bangkok 10220, Thailand; E-Mail: narong_1960@hotmail.com

**Keywords:** dihydrochalcone, antiinflammatory activity, *Cyathostemma argenteum*, annonaceae

## Abstract

A new dihydrochalcone derivative, 4',6'-dihydroxy-2',4-dimethoxy-5'-(2''-hydroxybenzyl)dihydrochalcone (**1**) and one known dihydrochalcone, 4',6'-dihydroxy-2',4- dimethoxydihydrochalcone (**2**) were isolated from leaves and twigs of *Cyathostemma argenteum*. Their structures were established by spectral methods, mainly 2D NMR spectroscopic techniques, which involved combined applications of DEPT, COSY, HMQC and HMBC. The molecular structure of **1** was also confirmed by single crystal X-ray diffraction. The test compounds **1** and **2** displayed significant inhibitory activity at a dose of 1 mg/ear on edema formation at all determination times, with similar intensity as phenylbutazone.

## 1. Introduction

**Figure 1:**
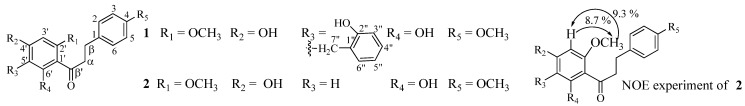
Structures of isolated compounds **1**–**2**.

*Cyathostemma argenteum* is one of the six known *Cyathostemma* species (*argenteum*, *longipes*, *micranthum*, *siamensis*, *viridiflorum*, *wrayi*) belonging to the family Annonaceae [1]. It is a flowering and woody vine plant which is widely grown in Thailand. Previous bioactivity investigations of the *Cyathostemma* genus showed that the methanolic extract from root and stem bark of this plant were effective against breast cancer [1,2]. Although it is known for producing a variety of secondary metabolites including alkaloids [1,3]; flavonoids [3] and shikimic acid derivatives [4,5], the constituents of this plant have not been studied much. Within the scope of our continuous search for bioactive compounds from natural plants, the leaves and twigs of *C. argenteum* were further investigated. In this paper, we report the first isolation and structural elucidation of one novel dihydrochalcone 1 along with another previously known chalcone derivative 2 (Figure 1). In addition, the compounds were evaluated for their *in vivo* antiinflammatory activity. 

## 2. Results and Discussion

Compound **1** crystallized as white needles which gave a [M+Na]^+^ quasimolecular ion peak at *m/z* 431.15729 in the HRESIMS spectrum, consistent with the molecular formula C_24_H_24_O_6_. The peaks in the EIMS at *m/z* 246 (100%) resulting from cleavage of a cinnamoyl moiety revealed the existence of a dihydrochalcone skeleton with a methoxy-substituted ring B and a ring A substituted with three hydroxyls, one methoxy and one benzyl group. The UV spectrum of **1** showed absorption maxima at 240 and 295 nm, suggesting the presence of a chalcone [6]. The IR spectrum showed bands at 3150 and 1616 cm^−1^ that were attributed to chelated carbonyl and phenolic hydroxyl groups, respectively. The ^1^H-NMR spectrum of **1** (Table 1) showed two methylene triplets at *δ* 3.28 and 2.90 with a coupling constant of 7.7 Hz in agreement with H_2_-α and H_2_-β, respectively. The multiplet signals at *δ* 7.18 and obscure signals at *δ* 6.83 Hz were assigned to the four aromatic protons of ring B, H-2, H-6 and H-3, H-5, respectively, thus the methoxy group was located at the C-4 position. Additionally, the HMBC spectrum of **1** showed correlations between H-β and C-1, C-6, C-α, C-β', H-α and C-1, C-2, C-6, C-β, C-β', H-3 and C-1, C-2, C-3, C-4, C-5, H-2 and C-1, C-3, C-4, C-6, C-β, H-4(OCH3) and C-4 (Table 2).

**Table 1:** ^1^H-NMR (400 MHz), ^13^C-NMR (100 MHz) in acetone-d_6_ data for the isolated dihydrochalcone compounds **1** and ^1^H-NMR (500 MHz), ^13^C-NMR (125 MHz) for **2** in CDCl_3_.

Position	4',6'-Dihydroxy-2',4-dimethoxy-5'-(2''-hydroxybenzyl)dihydrochalcone (1)		Position	4',6'-Dihydroxy-2',4-dimethoxydihydrochalcone (2)	
	* δ ^1^H (J Hz)	δ ^13^C (DEPT)		* δ ^1^H (J Hz)	δ ^13^C (DEPT)
1	-	133.64 (C)	1	-	133.72 (C)
2	7.18 m	129.30 (CH)	2	7.18 m	129.37 (CH)
3	6.83 obsc.	113.70 (CH)	3	6.87 dd (2.07, 6.6)	113.87 (CH)
4(OCH3)	3.75 s	158.07 (C)	4(OCH3)	3.82s	157.88 (C)
5	6.83 obsc.	133.70 (CH)	5	6.87 dd (2.07, 6.6)	113.87 (CH)
6	7.18 m	129.30 (CH)	6	7.18 m	129.37 (CH)
α	3.28 t (7.7)	45.89 (CH2)	α	3.31 t (7.8)	46.08 (CH2)
β	2.90 t (7.7)	29.75 (CH2)	β	2.96 t (7.8)	29.81 (CH2)
β'	-	205.40 (C)	β'	-	204.68 (C)
1'	-	104.87(C)	1'	-	105.95 (C)
2′(OCH3)	3.86s	161.81 (C)	2'(OCH3)	3.87s	162.31 (C)
3'	6.13s	91.01 (CH)	3'	5.93 d (2.4)	90.64 (CH)
4'(OH)	-	162.26 (C)	4'(OH)	5.56 br s	163.48 (C)
5'	-	106.93(C)	5'	6.02 d (2.4)	96.60 (CH)
6'(OH)	14.76s	164.70 (C)	6'(OH)	13.97s	167.34 (C)
1''	-	126.99 (C)	4(OCH3)	-	55.27 (CH3)
2''(OH)	-	154.13 (C)	2′(OCH3)	-	55.68 (CH3)
3''	6.83 obsc.	115.05 (CH)			
4''	7.01ddd (1.6, 7.5, 7.7)	126.95 (CH)			
5''	6.74 ddd (1.6, 7.5, 7.7)	119.85 (CH)			
6''	7.23 dd (1.6, 7.5)	130.39 (CH)			
7''	3.89s	21.87 (CH2)			
4(OCH3)		54.52 (CH3)			
2'(OCH3)		55.19 (CH3)			

* δ in ppm from TMS [coupling constants (J) in Hz are given in parentheses]; obsc. = obscure signal.

**Table 2:** ^1^H-^13^C, ^1^H-^1^H correlations for isolated dihydrochalcone derivatives.

Position H	4',6'-Dihydroxy-2',4-dimethoxy-5'-(2''-hydroxybenzyl)-dihydrochalcone (1)		Position H	4',6'-dihydroxy-2',4-dimethoxy-dihydrochalcone (2)	
	HMBC Correlation	COSY Correlation		HMBC Correlation	COSY Correlation
1	-	-	1	-	-
2	C-1, 3, 4, 6, β	H-3	2	C-1, 3, 4, β	H-3
3	C-1, 2, 3, 4, 5	H-2	3	C-1, 2, 4, 5	H-2
4(OCH_3_)	C-4	-	4(OCH_3_)	C-4	-
5	C-1, 3, 4, 6	H-6	5	C-1, 3, 4	H-6
6	C-1, 2, 4, 5, β	H-5	6	C-1, 2, 4, 5, β	H-5
α	C-1, 2, 6, β, β′	H-α, β	α	C- 1, β, β′	H-α, β
β	C-1, 6, α, β′	H-α, β	β	C- 1, 2, 6, α, β'	H-α, β
β'	-	-	β'	-	-
1'	-	-	1'	-	-
2'(OCH_3_)	C-2'	-	2′(OCH3)	C- 2'	-
3'	C-1', 2', 4', 5', β′	-	3'	C- 1', 2', 4', 5'	-
4'	-	-	4'(OH)	-	-
5'	-	-	5'	C-1', 3'	-
6'(OH)	C-1', 5', 6', 7''	-	6'(OH)	C-1', 5', 6'	-
1''	-	-	4(OCH3)	C-4	-
2''	-	-			
3''	C-1'', 2'', 4'', 5''	-			
4''	C-2'', 3'', 5'', 6''	H-5'', 6''			
5''	C-1'', 3'', 4'', 6''	H-4'', 6''			
6''	C-1'', 2'', 4'', 5'', 7''	H-5'', 4''			
7''	C-4', 5', 6', 1'', 2'', 6''	H-7''			
4(OCH3)	C-4	-			

**Figure 2:**
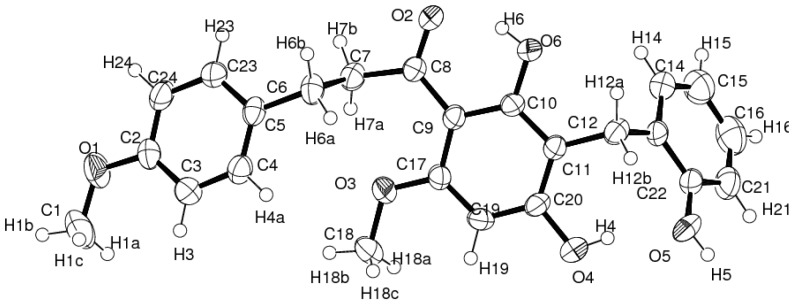
ORTEP view of the asymmetric unit of **1** with the atom-labelling scheme, showing 50% probability displacement ellipsoids.

The chelated hydroxy proton signal of ring A appeared at *δ* 14.76 and a resonance at *δ*_C_ 205.40 for a chelated carbonyl group were consistent with a 6'-hydroxychalcone. Furthermore, the methoxy (*δ*_H_ 3.68, *δ*_C_ 55.19), H-3' (*δ* 14.76, s) and a benzyl group substituents were established from NMR (Table 1) and MS data. From the biosynthetic point of view the remaining methoxy and hydroxy groups should be attached to C-2' and C-4', respectively. The ^1^H-NMR spectrum also showed features of hydroxybenzyl moiety (*δ* 3.89 assigned to a singlet of magnetically equivalent methylene protons), and signals for four aromatic protons, H-3'' *δ* 6.83 obsc., H-4'' *δ* 7.01 ddd (1.6, 7.5, 7.7), H-5'' *δ* 6.74 ddd (1.6, 7.5, 7.7), H-6'' *δ* 7.23 dd (1.6, 7.5). The HMBC spectrum showed the correlation between H-6′(OH) and C-7'' confirming the hydroxybenzyl group to be connected at C-5'. Moreover, the pattern of substitution on the hydroxybenzyl ring was again confirmed using HMBC spectra. The H-7'', methylene proton showed HMBC correlations to C-1'', C-2'' and C-6''. On the other hand, the splitting pattern of the aromatic proton H-4'', H-5'', H-6'' and HMBC correlation (Table 1 and Table 2) also confirmed presence of the *o*-hydroxybenzyl group. Thus, the structure of compound **1** was determined as 4',6'-dihydroxy-2',4-dimethoxy-5'-(2″-hydroxybenzyl)dihydrochalcone. The dihydrochalcone is one of the major classes of secondary metabolites in the plant kingdom and is biosynthesized *via* both the shikimate and acetate pathways. The biosynthesis of this compound requires *p*-dihydroxycoumaroyl-CoA as a precursor. It is assumed the biosynthesis may involve interconversion between *p*-coumaroyl-CoA and *p*-dihydrocoumaroyl-CoA catalyzed by NADPH-dependent dehydrogenase. In fact, a dihydrochalcone such as compound **1**, must have two nucleophilic centers at the hydroxyl group and the *ortho*-C-3', C-5' position in the A-ring to perform a nuclephilic substitution reaction with *o*-hydroxybenzoyl-CoA. Thus, the *o*-hydroxybenzyl group of compound **1** was confirmed at C-5' position which corresponded to the biosynthesis pathway [7]. This structure was also confirmed by X-ray crystallographic analysis (Figure 2). 

**Figure 3:**
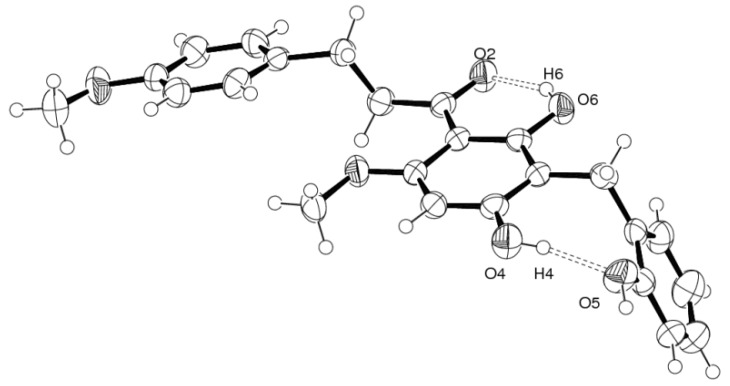
ORTEP drawing of strong intramolecular O–H…O hydrogen bonds in **1**.

**Figure 4:**
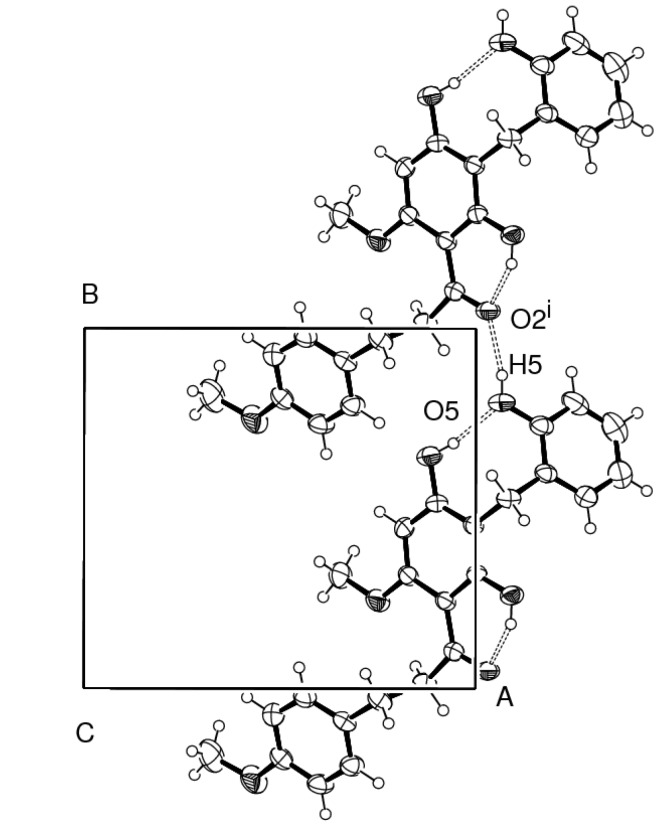
ORTEP drawing of strong intermolecular O–H…O hydrogen bond in **1**.

Compound **1** presented one crystallographically independent molecule in the asymmetric unit. The molecule is twisted with a dihedral angle of 16.70(11)° between benzene rings A and B which is significantly different to that reported in [8,9] due to not only to the substituted hydroxybenzyl ring (ring C) at C-12 of ring A with dihedral angle of 70.23(10)°, but also the influence of the strong intramolecular O-4–H-4…O-5 hydrogen bond interaction of the substituted 4′-and 2″-hydroxyl groups which are generated as a *S*(8) motif. In addition, the 4′-hydroxyl group at C-20 is not coplanar compared to one hydroxyl and one methoxy substituent in the A ring as well as the methoxy substituent on ring B that are closely coplanar, especially the strong intramolecular O-6–H-6…O-2 hydrogen bond produced the *S*(6) motif as observed in [8] with six non-hydrogen *r.m.s.* 0.0112(1) and 0.0056(1) Å, respectively. The torsion angle of the 1-propane unit [C-6–C-7–(C-8=O-2)] attached to rings A and B is 95.49(1)° and dihedral angles of 66.40(2) and 83.16(2)° are observed (Figure 3 and Table 3). Figure 4 shows the crystal packing of **1** which generated 1-D supramolecular hydrogen bond chains along the [001] plane.

**Table 3:** The selected strong hydrogen bond interactions in **1**.

**D–H…A**	**d[D–H] (Å)**	**d[H…A] (Å)**	**d[D…A] (Å)**	**∠[D–H…A] (°)**
O-4–H-4…O-5	0.820	1.856	2.662(3)	168
O-5–H-5…O-2 ^i^	0.820	1.903	2.704(2)	165
O-6–H-6…O-2	0.820	1.743	2.477(2)	148

Symmetry code (i) x,y-1,z.

Compound **2** was isolated as pale yellow needles. Its molecular formula, C_17_H_18_O_5_ was obtained from analysis of the HRESIMS ([M+Na]^+^), *m/z* 325.11542 and NMR spectroscopic data. General analysis of the NMR spectroscopic data (Table 1 and Table 2) showed that its spectrum resembled that of compound **1**, except for the resonance of its hydroxybenzyl group. The IR absorption frequencies (3258, 1643 cm^−1^) of the hydroxyl and carbonyl groups indicated their involvement in intramolecular hydrogen-bonding. In addition, the ultraviolent spectrum λ_max_ (log *ε*): 4.18 (289) nm supported the presence of a conjugated ketone in the structure. The ^1^H-NMR spectrum of **2** showed signals for a chelated and normal phenolic hydroxy function (*δ* 13.97s and 5.56 br s), two aromatic methoxy groups (*δ* 3.82s and 3.87s), and six aromatic protons, as in **2**. Four of these aromatic protons appeared as a doublet of doublets of H-3, 5 [(*δ* 6.87 dd (2.07, 6.6)] and a multiplet for H-2, 6 (*δ* 7.18), representing an AB system of a *p*-disubstitued benzene derivative. The remaining two aromatic protons of **2** also appeared as a pair of doublets [(*δ* 5.93 and 6.02 (*J* = 2.4 Hz)] corresponding to two *meta*-coupled protons, which resembled those of H-3′ and H-5′ of **2**. However, the spectrum of **2** showed two two-proton triplets at *δ* 3.31 and 2.96 (*J* = 7.8 Hz) typical of H-α and H-β of a dihydrochalcone derivative [8]. The distributions of the methoxy and hydroxy groups in the two benzene rings of **2** were confirmed by the mass spectral fragmentation of the compound. The mass spectrum showed a base peak at *m/z* 134 (100%) indicating the presence of a methoxy group in the ring B, whereas the intense peak at *m/z* 167 (84) suggested that ring A contains both methoxy and hydroxy groups. The identification of H-3' in ring A was determined by NOE difference spectra. Irradiation of H-3' signal showed enhancement of the methoxy group, thus indicating that it definitely has one methoxy next to H-3'. In addition, irradiation at the methoxy signal of **2** also showed enhancement of H-3'. On the other hand, the H-5' was confirmed at this position since there was no NOE enhancement with the methoxy group and HMBC correlations technique with H- 6'(OH). From the foregoing spectroscopic data, the structure of **2** was deduced to be 4',6'-dihydroxy-2',4-dimethoxydihydrochalcone, which is identical to that previously assigned to a chalcone derivative isolated from the leaves of *Rauwenhoffia siamensis* Scheff [9]. 

The results of the present study also revealed the anti-inflammatory activity of both tested compounds in the acute phase of inflammation (Table 4). Formation of EPP-induced rat ear edema is a useful model for screening and investigating the anti-inflammatory activity of test compounds in this phase of inflammation. The inflammatory mediators released in this model include histamine, serotonin, bradykinin and prostaglandin (PGs). These mediators are capable of promoting vasodilation and increasing vascular permeability as well as synergistically producing edema [10]. 

**Table 4:** Antiinflammatory activity of compounds **1** and **2**.

Group	Dose (mg/ear)	Edema thickness ( m)				% edema inhibition			
		15 min	30 min	1 h	2 h	15 min	30 min	1 h	2 h
Control (Acetone)	-	160.00 ± 7.30	180.00 ± 5.16	203.33 ± 8.03	193.33 ± 4.94	-	-	-	-
Phenylbutazone	1	30.00 ± 13.41 *	43.33 ± 9.54 *	73.33 ± 12.29 *	90 ± 4.47 *	81.25	75.93	63.93	53.45
**Compound 1**	1	16.67 ± 2.11 *	40.00 ± 5.16 *	76.67 ± 8.03 *	83.33 ± 3.33 *	89.58	77.78	62.30	56.90
**Compound 2**	1	1.67 ± 1.67 *	23.33 ± 2.11 *	63.33 ± 4.22 *	66.67 ± 8.82 *	98.96	87.04	68.85	65.52

Results are expressed as mean ± S.E.M. (N of ears = 6); Significantly different from the control group: * *p* < 0.05.

It was found that both test compounds **1** and **2** elicited a significant inhibitory effect on the edema formation at all assessment times at the dose of 1 mg/ear, similar to that of phenylbutazone. It is suggested that test both compounds probably possess anti-inflammatory activity by inhibition of the release or synthesis of various inflammatory mediators.

## 3. Experimental

### 3.1. General

^1^H and ^13^C-NMR, ^1^H-^1^H COSY, HMQC and HMBC spectra were recorded with a Unity *plus* 500 spectrometer (Varian Inc, Palo Alto, CA, USA) operating at 500 MHz for ^1^H, and 125 MHz for ^13^C, respectively. ^1^H and ^13^C-NMR spectra were also measured with a DPX on Bruker operating at 400 MHz for ^1^H, and 100 MHz for ^13^C, respectively. Low resolution mass spectra were recorded on a Thermo Finnegan Polaris Q mass spectrometer at 70 eV (probe) for EIMS. HRESIMS was obtained by using a Finnigan LC-Q Advantage Thermoquest spectrometer equipped with Xcalibur software (both intruments from Thermo Finnigan, Waltham, MA, USA). UV spectrum was measured on a Shimadzu 1601 spectrophotometer (Shimadzu, Kyoto, Japan). IR spectra in KBr disk were recorded on Shimadzu 8900 FTIR spectrophotometer. The X-ray data set was collected at 296(2)K on a X8 APEX II diffractometer (Bruker AXS Inc, Karlsruhe, Germany), using Mo-Kα radiation (λ = 0.71073 Å). Melting points were recorded in degree Celsius (°C) and were measured on a digital Electrothermal melting apparatus. Column chromatography was conducted on silica gel 60 (Merck 7734, 70–230 mesh). TLC was performed on aluminium backed pre-coated silica gel 60 PF_254_ sheets and detection with using UV detector.

### 3.2. Plant Material

The leaves and twigs of *C. argenteum* (Annonaceae) were collected in October 2011 from a swamp forest in Ubon Ratchathani Province, Thailand. The species was identified by Mr. Narong Nantasean, from The Forest Herbarium, Department of National Park, Wildlife and Plant Conservation, Ministry of Natural Resources and Environment, Bangkok, Thailand. A voucher specimen (BKF.18053) was deposited in the herbarium of this institute.

### 3.3. Extraction and Isolation

The dried, powdered leaves and twigs of *C. argenteum* (1.85 kg) were successively macerated without agitation at room temperature for 72 h in hexane (3 × 12 L) and 2:1 EtOAc/MeOH (3 × 12 L). Evaporation of the hexane and EtOAc/MeOH gave a pale yellow amorphous solid (28 g) and a dark brown solid (111 g), respectively. A portion of the EtOAc/MeOH extract was chromatographed over a column of silica gel (Merck Art 7734, 350 g) with hexane/acetone/MeOH to give 10 fractions F1-F10. Subfraction F8 (2.05 g) was rechromatographed over a column of silica gel with hexane/acetone/MeOH and crystallized using EtOH to furnish compound **1** (256 mg). Fraction F5 (3.11 g) was rechromatographed over silica gel using a hexane-EtOAc gradient, to give three subfractions A1–A3. The pale yellow subfraction A1 (1.06 g) was repeatedly recrystallized from EtOH to afford compound **2** (170 mg). Compounds **1**–**2** were subjected to an antiinflammatory activity test by the ear edema model, according to Brattsand *et al.* [11].

### 3.4. Spectroscopic Data

*4′,6′-Dihydroxy-2′,4-dimethoxy-5′-(2″-hydroxybenzyl)dihydrochalcone* (**1**): White needles. Mp 158.3–159 °C. UV (EtOH) λ_max_(log *ε*): 3.48 (295), 3.07 (240) nm. IR (KBr) *ν*_max_ 3150, 2937, 1616, 1580, 1568, 1512, 1454, 1416, 1330, 1265, 1252, 1196, 1151, 1101, 1130, 827, 756 cm^−1^. ^1^H-NMR (Acetone-d_6_, 500 MHz) data see Table 1,^13^C-NMR (CDCl_3_, 125 MHz) data see Table 1. EI-MS *m/z*: 408 [M]^+^(61), 390(19), 377(15), 283(16), 273(24), 264(100), 180(28), 179(82), 153(39), 121(27), 91(10), 77(11). HR-ESI-MS (pos.) *m/z*: 431.15729 ([M+Na]^+^, C_24_H_24_O_6_Na. calcd. 431.15732).

*4′,6′-Dihydroxy-2′,4-dimethoxydihydrochalcone* (**2**): Pale yellow needles. Mp 172–172.9 °C. UV(EtOH) λ_max_(log *ε*): 4.18 (289) nm. IR (KBr) *ν*_max_ 3258, 3020, 2952, 2833, 1643, 1630, 1610, 1568, 1512, 1417, 1436, 1296, 1244, 1196, 1164, 1111, 1032, 817 cm^−1^. ^1^H-NMR (CDCl_3_, 400 MHz) data see Table 1, ^13^C-NMR (CDCl_3_, 100 MHz) data see Table 1. EI-MS *m/z*: 302 [M]^+^(44), 283(44), 167(84), 134(100), 121(29), 91(12), 77(8). HR-ESI-MS (pos.) *m/z*: 325.11542 ([M+Na]^+^, C_17_H_18_O_5_Na. calcd. 325.11551).

### 3.5. X-ray Crystallographic Analysis of **1**

Molecular formula C_24_H_24_O_6_, *M*r = 408.43, monoclinic, *Pc*, *a* = 11.1360(6) Å, *b* = 10.2061(6) Å, *c* = 8.9779(4) Å, *β* = 94.756(3)°, *V* = 1016.87(9) Å^3^, *Z* = 2, *D*c = 1.334 Mg/m^3^, *μ* = 0.096 mm^−1^, *T* = 296(2) K. Three thousand four hundred and ten reflections (3226 independent, *R*_int_ = 0.000) were collected in *θ* range from 1.84 to 25.73°. Largest electron density residue: 0.119 e.Å^−3^, *R*_1_ (for *I* > 2*σ*(*I*)) = 0.0349 and *wR*_2_ = 0.0937 (all data) with *R*_1_ = ∑||*F*_o_| –|*F*_c_||/∑|*F*_o_| and *wR*_2_ = ∑*w*(*F*_o_^2^–*F*_c_^2^)^2^/∑*w*(*F*_o_^2^)^2^)^0.5^. The structure was solved by direct methods using *SHELXS-97* [6] and all non-hydrogen atoms were refined anisotropically using the least-squares method on *F*^2^ using *SHELXL-97* [12]. All the H atoms in these compounds were calculated geometrically with isotropic displacement parameters set to 1.2 (1.5 for hydroxyl and methyl groups) times the equivalent isotropic *U* values of the parent carbon atoms. The molecular graph was illustrated using *ORTEP* [13]. The crystallographic data for the structure of **1** reported in this paper has been deposited with the Cambridge Crystallographic Data Centre as supplementary publication (Deposition No. CCDC 937638). This data can be obtained free of charge from *via* www.ccdc.cam.ac.uk/conts/retrieving.html (or from the CCDC, 12 Union Road, Cambridge CB2 1EZ, UK; fax: +44 1223 336033; e-mail: deposit@ccdc.cam.ac.uk).

### 3.6. Antiinflammatory Activity: Ethyl Phenylpropiolate (EPP)-induced Ear Edema in Rats

The method of Brattsand *et al.* [11] was used. *Animals*: Male Sprague–Dawley rats weighing 40–60 g purchased from the National Laboratory Animal Center, Nakorn Pathom Province, Thailand, were used. All animals were kept in a room maintained under environmentally controlled conditions of 24 ± 1 °C and 12 h light–12 h dark cycle. The animals had free access to water and food. They were acclimatized at least 1 week before starting the experiments. *Testing*: Ear edema was induced by the topical application of either EPP dissolved in acetone to the inner and outer surfaces of both ears by means of an automatic microliter pipet. Test compounds **1** and **2**, at the dose of 1 mg/ear, were dissolved in acetone and applied topically in a volume of 20 μL to the inner and outer surfaces of the ear just before the irritants. The control group received acetone. All experiments were approved by Animal Ethics Committees, Faculty of Medicine, Chiang Mai University, Thailand. *Statistical analysis*: All data were expressed as mean ± S.E.M. Statistical comparison between groups was analyzed by using one-way analysis of variance (ANOVA) and p values of less than 0.05 were considered significant.

## 4. Conclusions

The new compound **4**',6'-dihydroxy-2',4-dimethoxy-5'-(2''-hydroxybenzyl)dihydrochalcone (**1**) was isolated from *C. argenteum*, together with one known dihydrochalcone **2**. Both compounds underwent additional biological screening for antiinflammatory activity. The experimental results suggest that both compounds probably possess anti-inflammatory activity by inhibition of the release or synthesis of various inflammatory mediators.
